# Hyperventilation Leading to Transient T-wave Inversion Mimicking Unstable Angina

**DOI:** 10.7759/cureus.12980

**Published:** 2021-01-29

**Authors:** Snehangsh Dash, Anil Kumar

**Affiliations:** 1 Internal Medicine, 12 Air Force Hospital, Gorakhpur, IND; 2 Cardiology, 7 Air Force Hospital, Kanpur, IND

**Keywords:** t-wave inversion, hyperventilation

## Abstract

T-wave inversion in ECG is very frequent and concerning finding as it is often associated with life-threatening conditions. There are numerous conditions mentioned in the literature for transient T-wave inversion such as acute coronary syndrome, cardiac memory T-wave, subarachnoid hemorrhage, electroconvulsive therapy, hyperventilation and indeterminate origin. Hyperventilation is already known as a cause of transient T-wave inversion; however, it is often forgotten in modern clinical settings. A 33-year-old doctor working in the same hospital reported to the emergency department during working hours with a history of acute onset breathing difficulties and atypical chest pain involving the retrosternal region. Arterial blood gas analysis (ABG) findings of respiratory alkalosis with transient T-wave inversion, which normalized soon after normal breathing and reassurance along with normal cardiac workup helped us to reach the correct diagnosis of hyperventilation syndrome.

## Introduction

T-wave inversion in ECG is very frequent and concerning finding as it is often associated with life-threatening conditions such as myocardial infarction or ventricular strain. However, there are various causes of T-wave inversion such as ischemic heart disease, metabolic disturbances, bradyarrhythmia, right ventricular hypertrophy, conduction disorders, changes during diagnostic coronary angiography, acute cerebral disease, acute adrenergic stress (Takotsubo cardiomyopathy), pulmonary oedema, antiarrhythmic drug effects, pulmonary embolism, cardiac memory secondary to transient tachycardia, post-ventricular pacing states and cocaine use [1]. There are numerous conditions mentioned in the literature for transient T-wave inversion such as acute coronary syndrome, cardiac memory T-wave, subarachnoid hemorrhage, electroconvulsive therapy, hyperventilation and indeterminate origin. Hyperventilation is already known as a cause of transient T-wave inversion; however, it is often forgotten in modern clinical settings [2]. Here we describe a case presented as breathlessness and atypical chest pain with transient T-wave inversion in ECG and diagnosed as hyperventilation and discharged with full recovery.

## Case presentation

A 33-year-old doctor working in the same hospital reported to the emergency department during working hours with a history of acute onset breathing difficulties and feeling of air hunger for half an hour duration along with a history of chest pain involving retrosternal region. There was no history of any fever, cough, anosmia, palpitation, syncope, audible wheeze, hemoptysis or autonomic symptoms. The doctor used to work in COVID ICU for a few days before the onset of symptoms. He does not consume tobacco in any form. He had a family history of hypertension and coronary artery disease.

**Figure 1:**
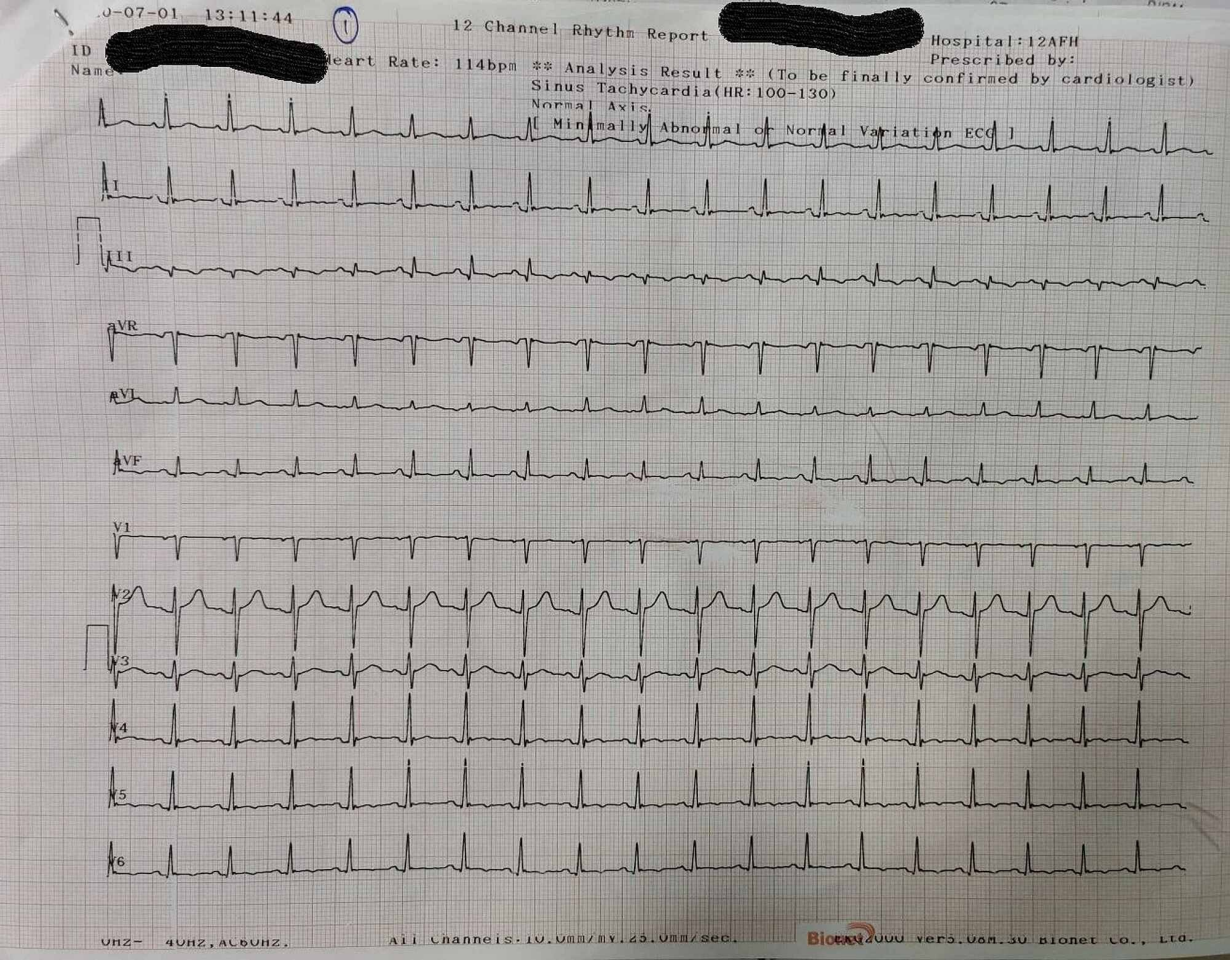
ECG on presentation

On initial examination, he appeared to be anxious. He was having tachycardia with heart rate of 110/min, his BP was 130/84 mmHg, he was afebrile and respiratory rate was 28/min. However, his oxygen saturation was 98% in room air but he was demanding supplemental oxygen. His systemic examination was normal. His ECG taken at emergency department showed tachycardia with T inversion in leads II, III, avF, V4-V6 (Figure 1). His arterial blood gas analysis (ABG) showed pH 7.52, pCO_2_ 20, pO_2_ 88, HCO_3_ 20, which was suggestive of respiratory alkalosis.

**Figure 2:**
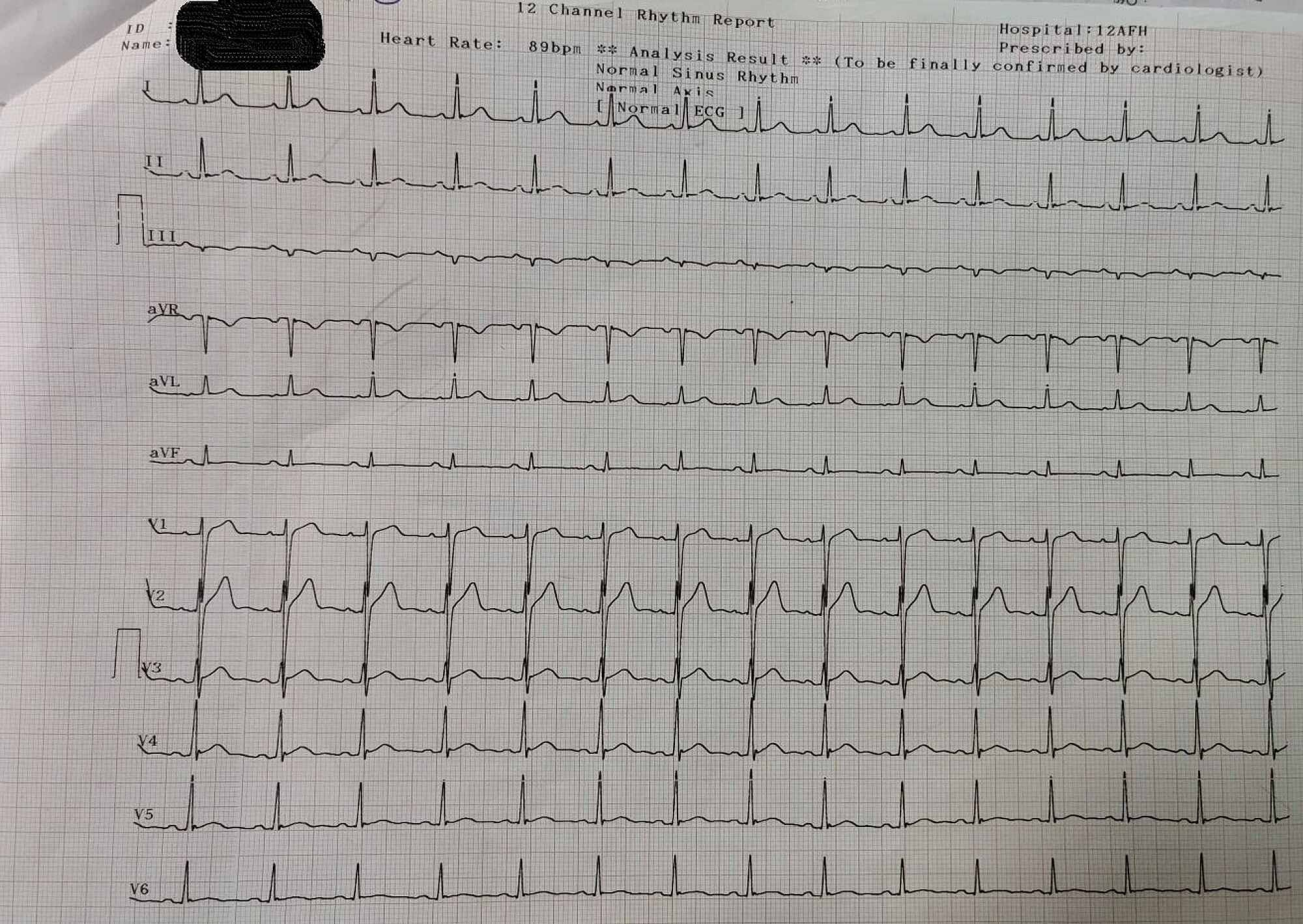
Repeat ECG after one hour after normal breathing and reassurance

**Figure 3:**
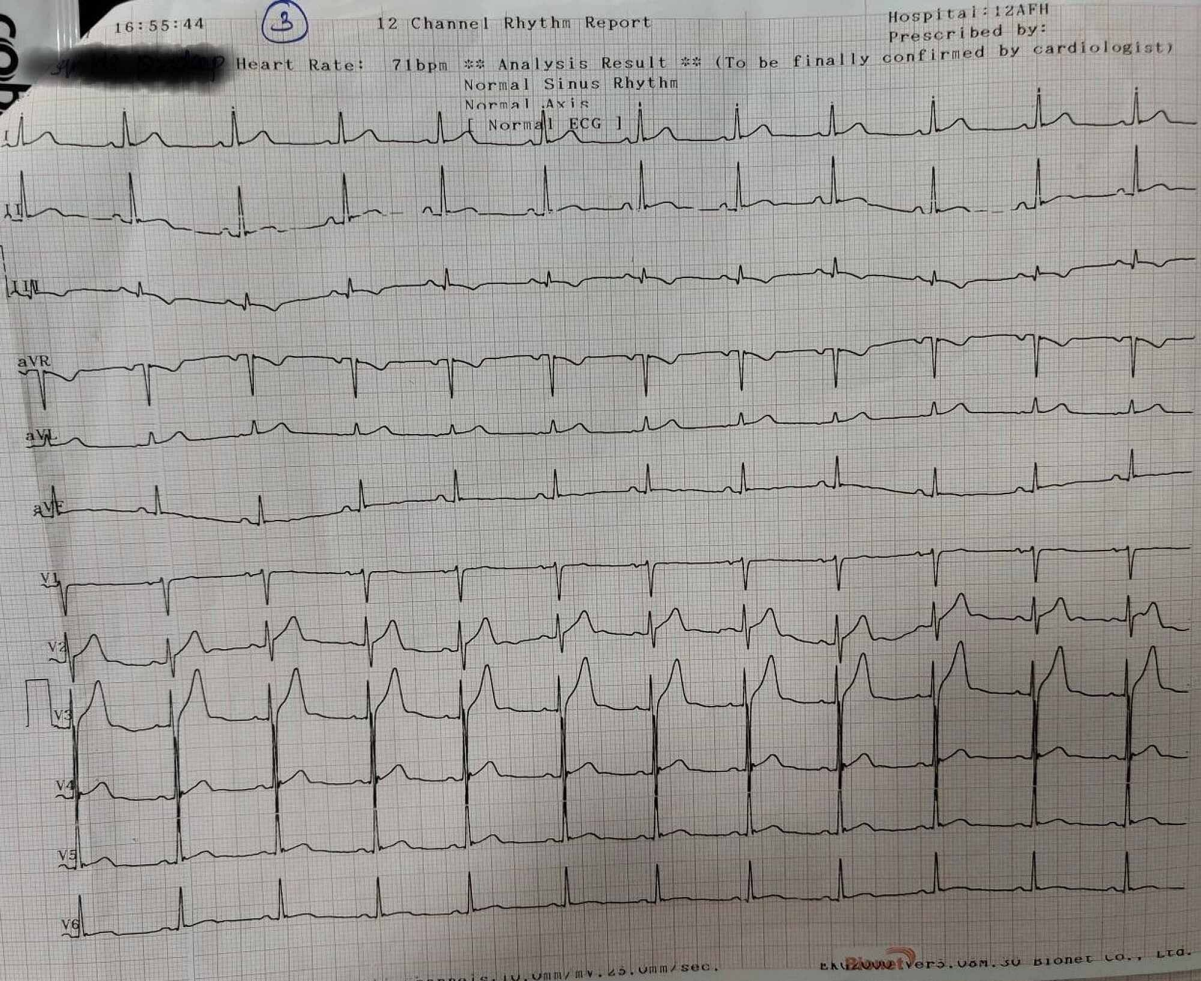
ECG prior to discharge

He was investigated further and hematological and biochemical investigations were normal. Trop-T was negative. Creatinine kinase myocardial band (CKMB) was normal. Chest X-ray was normal. Reverse transcription polymerase chain reaction (RT PCR) for COVID-19 was negative too. He was managed with reassurance and anti-anxiety measures. Repeat ECG after one hour was done and it revealed normal sinus rhythm with T inversion in only lead III (Figure 2). His old medical documents were perused and there was isolated T inversion in lead III in old ECGs. Further, he underwent 2D echo, 24-hour Holter monitoring, treadmill test, which were all normal. He was kept in ward for 24 hours' observation. Repeat ECG (Figure 3) showed no significant changes and he remained asymptomatic thereafter. He was discharged with complete recovery of symptoms and signs with a diagnosis of acute hyperventilation syndrome with transient T-wave inversion.

## Discussion

In leads I, II and V2 to V6, the T-wave is normally upright, inverted in lead aVR, and variable in leads III, aVL, aVF and V1. In general, an inverted T-wave in a single lead in one anatomic segment (i.e., inferior, lateral or anterior) is unlikely to represent acute pathology; for instance, a single inverted T-wave in either lead III or aVF can be a normal variant. The causes of inverted T-waves are variable. It can be a normal finding without pathological issues to clinical situations causing sudden death related to cardiac or respiratory syndromes.

Hyperventilation leading to an alteration in repolarization in ECG has been described [3,4]. T-wave inversion occurs in more than one lead due to hyperventilation and it gets corrected once hyperventilation is corrected [5,6]. The exact mechanism of T-wave inversion with hyperventilation is not understood yet, but it has been postulated that it is a direct result of hypocapnia. A study showed decreasing pCO_2_ reduced the amplitude of T-waves at fixed volume ventilation while separately increasing ventilation also decreased T-wave amplitude at a fixed pCO_2_ [7].

Another study involving 474 healthy participants showed that 15% developed repolarization changes in response to hyperventilation. The majority changes were T-wave inversion - either from positive to negative or negative to positive, e.g. lead III. Few of the subjects that did show repolarization abnormalities (13%) demonstrated ST depression. Most of the ST-T changes arose after brief (30-90 seconds) hyperventilation and soon after normal breathing returned to normal [5].

## Conclusions

Thus, in our case, ABG findings of respiratory alkalosis with transient T-wave inversion, which normalized soon after normal breathing and reassurance along with normal cardiac workup helped us to reach the correct diagnosis. Our patient had very low risk for coronary artery disease. The patients coming with dyspnea and low risk for coronary artery disease with ECG changes should undergo ABG to rule out hyperventilation and associated respiratory alkalosis.
